# Endoscopic full-thickness resection for a submucosal tumor at the esophageal entrance: is closure obligatory?

**DOI:** 10.1055/a-2748-1472

**Published:** 2025-12-08

**Authors:** Xiaoxiao Li, Jiyu Zhang, Miao Shi, Dan Liu

**Affiliations:** 1 191599 Department of Gastroenterology and Hepatology, The First Affiliated Hospital of Zhengzhou University, Zhengzhou, China

Creation of a tunnel has been deemed technically demanding for resecting large esophageal submucosal tumors (SMTs) originating from muscularis propria 1 . For lesions located at the esophageal entrance, which is known as the first physiological narrowing, there is no space to create a submucosal tunnel. As a result, endoscopic full-thickness resection (EFTR) is an imperative option 2 . Here, we present a large cervical esophageal SMT resected using EFTR, and the defect healing smoothly without closure.

A 51-year-old woman had a deep SMT at the esophagus entrance ( Fig. 1 **a** ). Endoscopic ultrasonography showed a well-demarcated, inhomogeneous and hypoechoic mass arising from the muscularis propria layer ( Fig. 1 **b** ). After acquiring informed consent, a standard EFTR was performed ( Video 1 ). To overcome the poor endoscopic maneuverability in this location, a snare was employed for traction to improve exposure and facilitate resection of the lesion while keeping the adventitia intact ( Fig. 1 **c** ). The resected specimen measured 25 mm × 18 mm ( Fig. 1 **d** ). Since the limited operating space made it challenging to close the defect, the procedure kept the esophageal adventitia intact ( Fig. 1 **e** ). The intact adventitia and soft tissues around esophageal entrance kept no air and liquid leakage. Thus, we decided to keep the defect unclosed. In addition, a gastric tube was placed in the esophageal lumen for decompression. On postoperative day 7, endoscopy showed no evidence of active bleeding or fistula formation; therefore, the gastric tube was withdrawn and the liquid diet was resumed. Four months later, endoscopic examination revealed satisfactory wound healing with no signs of esophageal stenosis and sphincter dysfunction ( Fig. 1 **f** ).

**Fig. 1:**
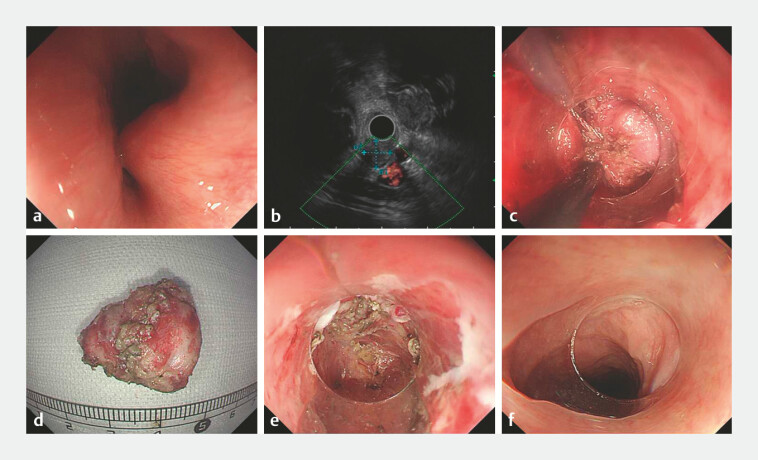
Endoscopic full-thickness resection for a large cervical esophageal submucosal tumor. **a** The deep submucosal tumor at the esophageal entrance. **b** Endoscopic ultrasonography showing a well-demarcated, inhomogeneous and hypoechoic mass arising from the muscularis propria layer. **c** Resection of the tumor with an IT knife and a snare as a contraction assistance. **d** The resected tumor specimen. **e** Endoscopic evaluation of the defect base. **f** Satisfactory wound healing 4 months postoperatively.

Endoscopic full-thickness resection for a submucosal tumor at esophageal entrance with no defect closure.Video 1

This case provides a feasible option that closure is not obligatory following EFTR at esophageal entrance, when the full-thickness defect is air tight.

Endoscopy_UCTN_Code_TTT_1AO_2AG_3AD

## Notes

Endoscopy E-Videos https://eref.thieme.de/e-videos
*E-Videos* is an open access online section of the journal *Endoscopy* , reporting on interesting cases and new techniques in gastroenterological endoscopy. All papers include a high-quality video and are published with a Creative Commons CC-BY license. Endoscopy E-Videos qualify for HINARI discounts and waivers and eligibility is automatically checked during the submission process. We grant 100% waivers to articles whose corresponding authors are based in Group A countries and 50% waivers to those who are based in Group B countries as classified by Research4Life (see: https://www.research4life.org/access/eligibility/ ). This section has its own submission website at https://mc.manuscriptcentral.com/e-videos .
